# Does rituximab improve clinical outcomes of patients with thyroid-associated ophthalmopathy? A systematic review and meta-analysis

**DOI:** 10.1186/s12886-018-0679-4

**Published:** 2018-02-17

**Authors:** Changjun Wang, Qingyao Ning, Kai Jin, Jiajun Xie, Juan Ye

**Affiliations:** Department of Ophthalmology, the Second Affiliated Hospital of Zhejiang University, College of Medicine, Hangzhou, 310009 China

**Keywords:** Inflammation, Orbit, Drugs, Eye lids, Vision

## Abstract

**Background:**

The current therapies of thyroid-associated ophthalmopathy (TAO) were still a challenging matter. In this study, we aimed to contrast the impact of before- after rituximab (RTX) therapy in the patients with TAO.

**Methods:**

We searched the PubMed, EMBASE, and SCOPUS databases for articles published up to July 3, 2017. Fixed- or random-effects meta-analysis was used to provide pooled estimates of standard mean difference (SMD) both the primary outcome from clinical activity score (CAS), and secondary outcomes from thyrotropin receptor antibody (TRAb), proptosis, thyroid stimulating hormone (TSH), and interleukin-6 (IL-6) levels. In addition, the quality and each study was assessed using either the Newcastle Ottawa Scale (NOS) or the Cochrane Risk of Bias tool, and reliability of the meta-analytic result using the Grading of Recommendations Assessment, Development and Evaluation (GRADE).

**Results:**

Of the 839 articles initially searched, 11 studies were finally eligible for inclusion. Subgroup analysis results showed that comparing with initial value, there was a decline in CAS at 1,3,6,12 month after RTX treatment, decreased TRAbs level at 6,12 month, proptosis improvement at least 1 month, unchanged IL-6 level at 6 month, decreased TSH level at 3 month but unchanged at 12 month. All included studies were classified as good quality.

**Conclusions:**

The pooled data suggested that the preliminary effects of RTX treatment on TAO might be promising. However, more large-sample and high-quality studies targeting RTX use during this disease and long-term surveillance of prognosis are urgently needed.

**Electronic supplementary material:**

The online version of this article (10.1186/s12886-018-0679-4) contains supplementary material, which is available to authorized users.

## Background

Thyroid-associated ophthalmopathy (TAO) is an autoimmune inflammatory orbital disease, which usually is caused by 20–50% of patients with Graves’ disease (GD) [1] and has the symptom of bilateral or unilateral eyeball protrusion, eyelid swelling, edema of periorbital tissue, and upper or lower eyelid retraction [2]. It was reported that lymphocytes B played an important role in TAO [3]. B cells could induce immune function via antigen presentation, co-stimulatory molecules expression and antibodies production. They can also be differentiated into antibody-producing plasma cells, which not only caused host defense, but also identified their own tissues, resulting in autoimmunity [4]. In addition, B cells produced cytokines that induced fbroblasts to generate glycosaminoglycan causing fluid and periorbital edema. In recent years, there have been many treatments for TAO, but adverse effects in follow-up should be cautious, such as hypertension, diabetes, stress ulcer, and osteoporosis during immunosuppressive therapies [5], retinopathy, neuropathy, and cataracts in single radiation therapy or combining with oral or intravenous steroids [6], around 20% to 25% of nonresponders after intravenous pulses of corticosteroids [7].

Rituximab (RTX) is a chimeric mouse monoclonal anti-human CD20 antibody against B-cell proliferation and maturation. RTX has been allowed since 2006 for treating rheumatoid arthritis and had a chance to become a candidate for the treatment of some other autoimmune diseases [8]. In recent years, although some case series have shown that RTX treatment in severe TAO may benefit patients, its application was restricted to case reports and uncontrolled studies, which was a lack of large-scale prospective studies and the effectiveness was still inconsistent. Therefore, we did a systematic review and aimed to check whether the CAS activity improved 1 month or more after RTX treatment for persons with TAO.

## Methods

### Search strategy

We conducted the systematic review according to the Preferred Reporting Items for Systematic Reviews and Meta-Analyses (PRISMA) statement [9]. To assess the evidence on this issue, a broad literature search was independently initiated by Changjun Wang and Qingyao Ning through the PubMed, EMBASE, and SCOPUS databases for articles published up to July 3, 2017 (Appendix 1). Search terms included the keyword terms using rituximab, Graves’ ophthalmopathy, thyroid associated orbitopathy, thyroid eye disease. Discrepancy was finally resolved by the senior author (Juan Ye). All literatures were retrieved with no language restrictions. If detailed data were available from online reports, we applied for it directly from authors. These literatures were managed and duplicates were removed through Endnote X7 software (Changjun Wang and Qingyao Ning).

### Study selection

The criteria deciding whether an article was included were as following: (1) cohort study or randomized controlled trial including RTX treatment for patients with TAO; (2) clinical data involving before-after RTX treatment would be available; (3) original articles including one or more parameters of CAS, TRAbs, proptosis, TSH, and IL-6 levels after clinical follow-up of at least 1 month. Studies would be removed if they were case reports, reviews, letters, or conference abstracts without full text.

### Data extraction and quality assessment

Kai Jin and Jiajun Xie extracted these data from each eligible article: authors’ names, the type of study, study period, country, age, gender, smoking status, follow-up. In this analysis, disease activity was estimated according to the seven-point CAS using Snellen chart based on the classical signs of inflammation (ocular pain, eyelid redness, eyelid swelling and fading eyesight) [10]. For each of included studies, CAS was calculated to examine the clinical improvement of these patients at the each ophthalmological visit. Instruments such as the Cochrane risk of bias tool for trials and the Newcastle-Ottawa Scale (NOS) for observational studies were also crosschecked by Changjun Wang and Kai Jin. Quality was assessed using the NOS and Cochrane and reliability of results using GRADE by Changjun Wang and Kai Jin [11, 12]. This was done in duplicate with disagreements handled by discussion with a final arbiter (Juan Ye).

### Statistical analysis

Based on the initial baseline value, the meta-analysis compared the outcome changes at different time interval (1,3,6,12 month, respectively) after RTX therapy, in view of mean CAS as the primary outcome, and mean of TRAbs, proptosis, TSH, IL-6 levels as secondary outcomes. Data were pooled and calculated their SMDs and the 95% confidence interval (CI) for each subgroup when at least two studies focused on the outcome (Additional file 1: Supplementary files). The meta-analysis was conducted by the Mantel–Haenszel method through Stata 12.0 software [13]. Statistical heterogeneity was estimated using the chi-square test *(χ*^*2*^*), τ*^*2*^ and *I*^*2*^ statistic. Where *p* < 0.05 or *I*^*2*^ > 50% appeared indicating moderate to large statistical heterogeneity, then a random-effects model was applied. We performed subgroup analysis for heterogeneity according to CAS, proptosis, TRAbs, TSH and IL-6. Otherwise, a fixed-effect model was performed. In addition, the impact of study quality on the results was evaluated by sensitivity analysis, and Egger’s tests were assessed for potential publication bias.

## Results

### Characteristics of eligible studies in the final analysis

**Fig. 1:**
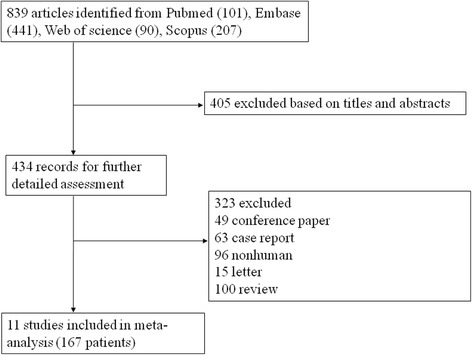
Study selection

Our initial search found 839 citations, and after review of title and abstract, 323 studies were recruited for further full-text reading, of which 11 articles were finally available for inclusion [14–24]. The selection flow was showed in Fig. 1. In this study, the mean age of patients was from 44.8 to 62.0 years old. A total of the population consisted of 49 (29.3%) men and 118 (70.7%) women, and almost one third smoked. Nearly one half of the participants were from China (44.9%), followed by Europe (31.7%) and North America (23.4%). The mean follow-up of these studies were from 1 to 60 months. Regarding adverse events, mild temperature elevation, nose and throat itching, headache, myalgias, optic neuropathy, orbital edema and decrease of vision, dyspepsia were reported (Table 1). Methodological quality in included studies were acceptable by Newcastle-Ottawa Scale and Cochrane risk of bias tool, in which main drawbacks were the application of few blinding and random distribution, and insufficient sample size, which were reliable appraised through GRADE. Then we tried to minimize it by subgroup analysis based on cohort study and randomized controlled trial, which indicated that most of these results were stable (Additional file 2: Table S1, Additional file 3: Table S2, Additional file 4: Figure S1, Additional file 5: Figure S2).

**Table 1:** Characteristic of the included studies in the meta-analysis

Author	Type of study	Study period	Country	Number of patients	Age (Mean ± SD) (Range)	Gender (M/F)	Smoking	Follow-up, Months (Mean ± SD)	Outcomes indexs	Complication
Salvi M et al. 2007 [14]	Cohort study	NA	Italy	9	44.8 ± 2.1 (31–51)	2/7	5	1	CAS, TRAbs, proptosis	3 nose and throat itching,mild temperature elevation
Vannucchi G et al. 2010 [17]	Cohort study	NA	Italy	10	46.6 ± 2.2 (31–54)	2/8	NA	12	TRAbs, IL-6	NA
Silkiss RZ et al. 2010 [16]	Cohort study	2007.1–2010.10	United States	12	52.1 (34–80)	5/7	4	12	CAS, TSH	No
Khanna D et al. 2010 [15]	Cohort study	2007.10.1–2009.2.1	United States	6	54.3 ± 9.1	2/4	3	7.5 ± 6.4	CAS, proptosis	1 urinary tract infection;1 hypertension;1 cardiac arrest
Mitchell AL et al. 2013 [18]	Cohort study	2008–2012	UK	9	62 (37–87)	1/8	NA	16	CAS	2 headache;1 headache and chills without pyrexia;1 mild myalgia
Savino G et al. 2013 [19]	Cohort study	NA	Italy	5	48 ± 12.1	3/2	3	18	CAS, proptosis	2 nausea and temperature elevation
Erdei A et al. 2014	Cohort study	NA	Hungary	5	47.8 ± 12.2 (31–64)	1/4	4	60	CAS, TRAbs, TSH	NA
McCoy AN et al. 2014 [21]	Cohort study	2007.10.1-?	United States	8	57 ± 6.63	3/5	4	18	CAS	NA
Stan MN et al. 2015 [23]	Randomized controlled trial	NA	United States	13	57.6 ± 12.7	4/9	2	13	CAS, TRAbs, proptosis	2 myalgias;2 skin rash and itching);1 infectious;1 vasculitis;2 optic neuropathy;1 severe lacrimation;2 gastrointestinal;
Salvi M et al. 2015 [22]	Randomized controlled trial	NA	Italy	15	51.9 ± 13.1	1/14	10	19	CAS, TRAbs, TSH, proptosis	1 orbital edema and decrease of vision;1 hypotension;1 myocardial infarction
Li J et al. 2017 [24]	Randomized controlled trial	NA	China	75	48.1 ± 10.0	25/50	15	10	CAS, proptosis, IL-6	1 flushing;9 nose and throat itching;2 dyspepsia;9 temperature elevation

*NA* not available

### Heterogeneity test result and subgroup analysis

**Fig. 2:**
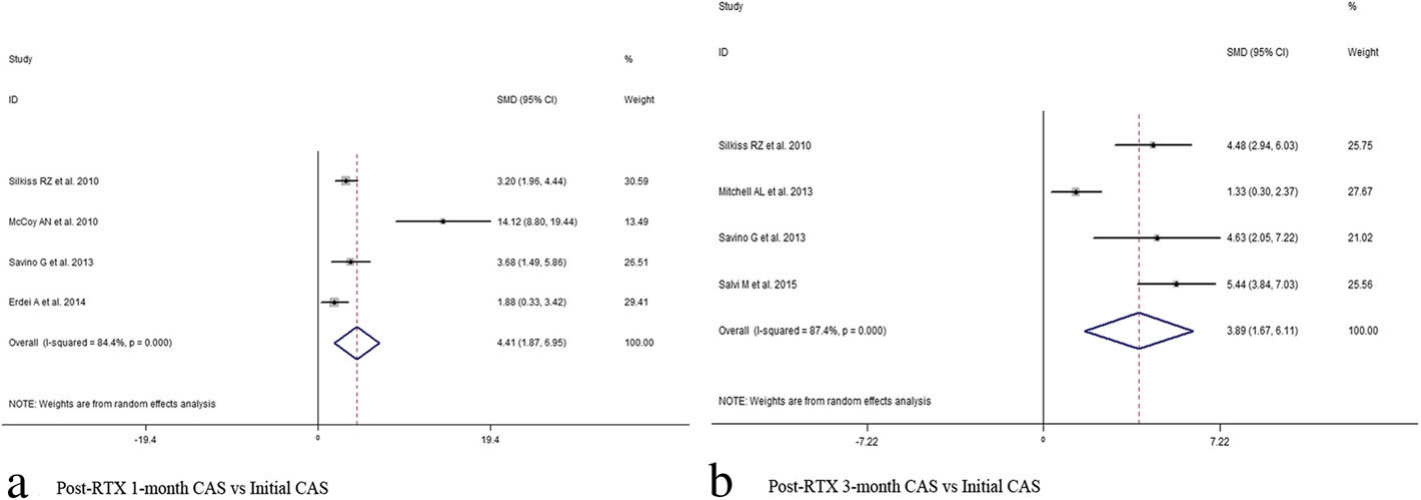
Comparing with initial value, there was a decline in CAS at 1 (**a**) and 3 (**b**) month after RTX treatment for TAO

**Fig. 3:**
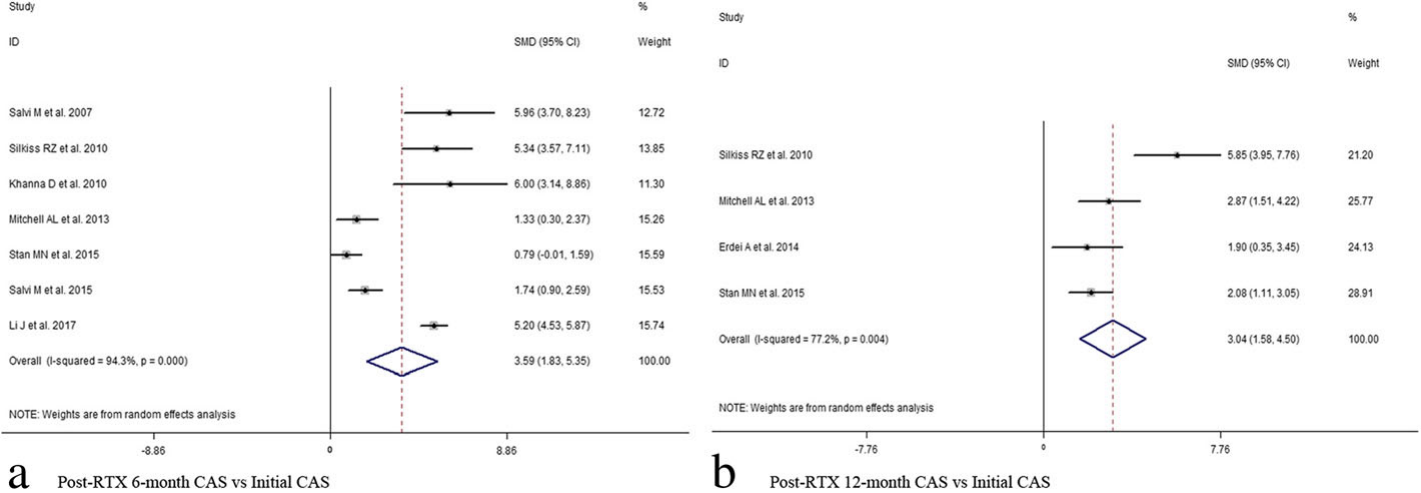
Comparing with initial value, there was a decline in CAS at 6 (**a**) and 12 (**b**) month after RTX treatment for TAO

**Fig. 4:**
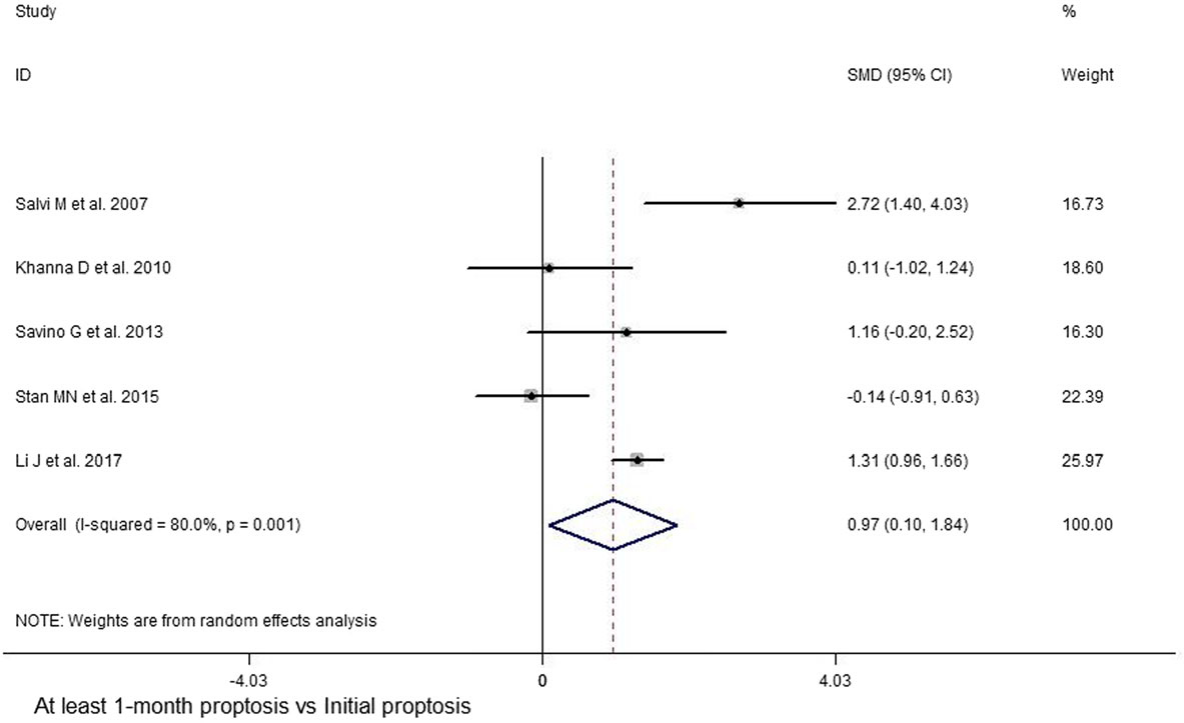
Comparing with initial value, there was a decrease in proptosis at least 1 month after RTX treatment for TAO

**Fig. 5:**
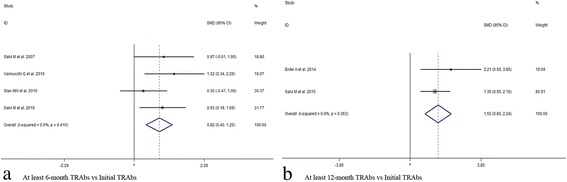
Comparing with initial value, there was a reduction in TRAbs at 6 (**a**) and 12 (**b**) month after RTX treatment for TAO

**Fig. 6:**
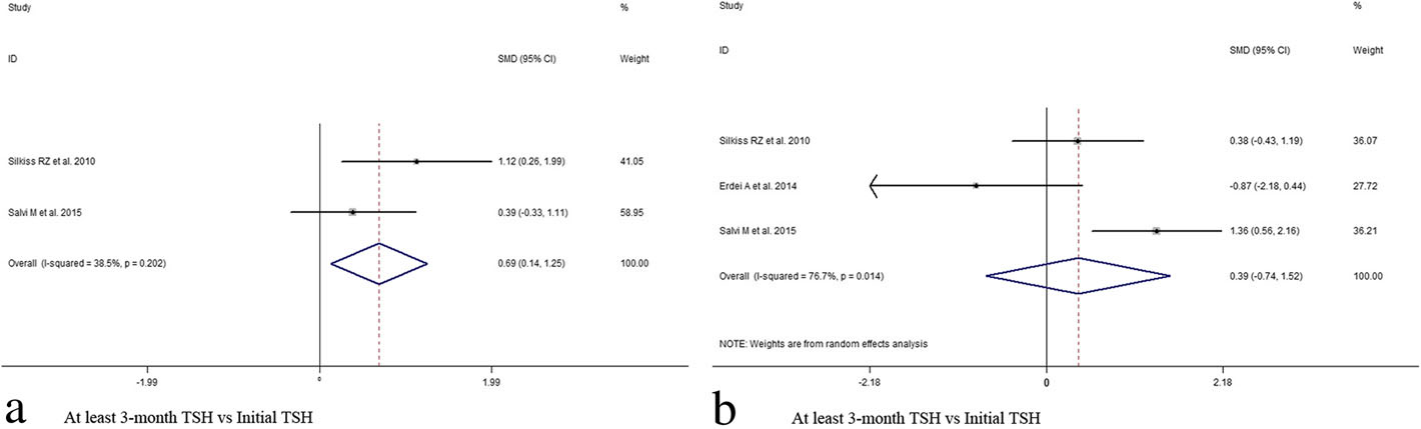
Comparing with initial value, there was a decline in TSH at 3 month (**a**) but unchangeable at 12 (**b**) month after RTX treatment for TAO

**Fig. 7:**
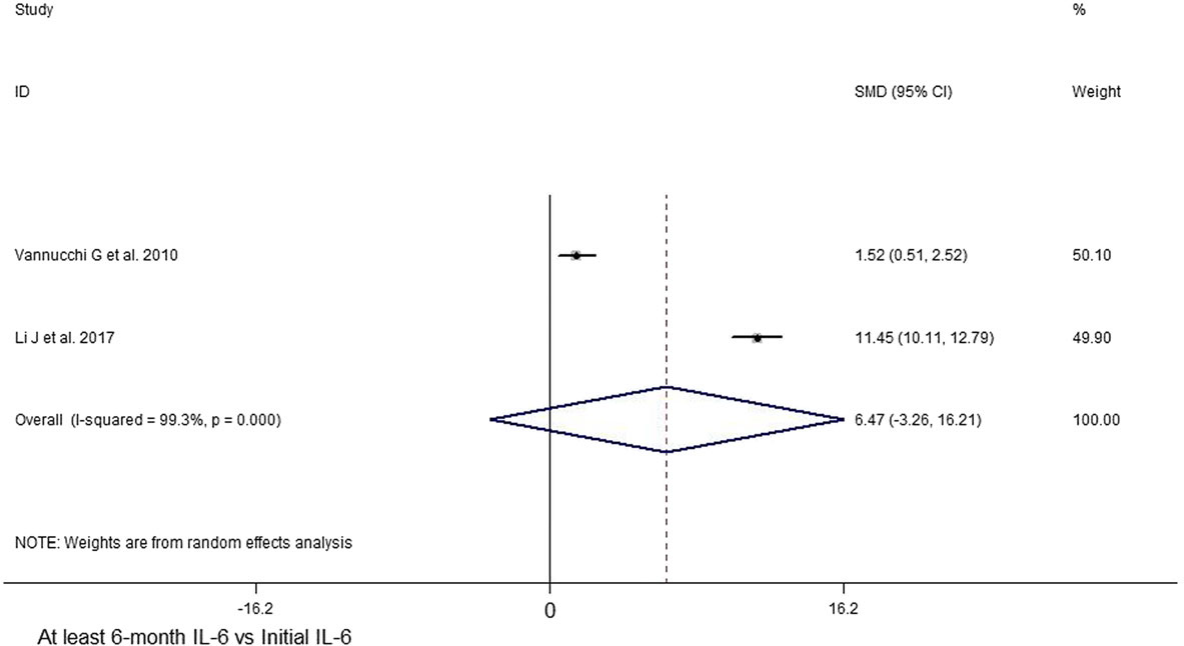
Comparing with initial value, there was unchangeable in IL-6 at 6 month after RTX treatment for TAO

In view of the initial CAS value, results indicated significant decrease at 1,3,6,12 months after subsequent RTX treatment (1 month, SMD 95% CI: 4.77 (2.77–6.77); 3 month, SMD 95% CI: 3.89 (1.67–6.11); 6 month, SMD 95% CI: 3.59 (1.83–5.35); 12 month, SMD 95% CI: 3.04 (1.58–4.50)) (Figs. 2 and 3, Additional file 6: Table S3). At last month of follow-up, proptosis improvement was shown (last month of follow-up, SMD 95% CI: 0.97 (0.10–1.84)) (Fig. 4). Similarly, pooled data reported TRAbs level were both declining at 6 and 12 months (6 month, SMD 95% CI: 0.82 (0.40–1.25); 12 month, SMD 95% CI: 1.52 (0.80–2.24)) (Fig. 5). TSH level was decreased at 2 month while unchanged at 12 month (2 month, SMD 95% CI: 0.69 (0.14–1.25); 12 month, SMD 95% CI: 0.39 (− 0.74–1.52)) (Fig. 6). In addition, IL-6 level was also not see an obvious variation (6 month, SMD 95% CI: 6.47 (− 3.26–16.21)) (Fig. 7). In Additional file 6: Table S3, subgroup analysis listed heterogeneity test using *I*^*2*^, in which the considerable heterogeneity (*I*^*2*^ > 75%) was dealed with random-effects model (CAS: 1,3,6,12 month: 84.4%, 87.4%, 94.3%,77.2%; proptosis: at least 1 month: 80%; TSH: 12 month: 76.7%; IL-6: 6 month: 99.3%).

### Sensitivity analysis and publication bias

We checked the effect of each of these studies through sensitivity analyses, and all were in accordance with our main results. Egger’s test suggested no publication bias was in this study (Additional file 6: Table S3).

## Discussion

Thyroid-associated ophthalmopathy was the main extrathyroidal manifestation of Graves’ disease. Treatment was depended on the evaluation of the activity and severity of TAO and focused on the patient’s living quality. In this systematic review and meta-analysis, we showed that RTX treatment may confer a favorable improvement against TAO. The improvement of TAO remained stable during the follow-up.

Regarding CAS, which may indicate whether these patients benefit or not from RTX therapy. Pain, redness, swelling and impaired function improved in most patients at 1 month after RTX treatment, and sustained during the 12-month follow-up. As shown in a previous study, there were evident reductions from the mean baseline CAS score 5.5 to finally approximate 1.0 for weeks 4, 8, 16, 24, 36, and 52 [16]. A high CAS could help select targeting patients who will benefit from RTX treatment. The eyelids became swollen and visual acuity decreased. The serious signs and symptoms in the baseline may result from the inflammation caused by the autoimmune course. Orbital fibroblasts were also considered to be vital in the pathogenesis [10]. Decreased CAS in the majority of individuals meant favorable therapeutic effects. The long-term reduction in CAS scores may suggest it may depend upon the late effects of the drug in some cases.

Recently, unchangeable proptosis was visible in Khanna et al. study [15]. It should be interpreted seriously because the study was only 6 patients and uncontrolled. But in this study, RTX therapy was effective in ameliorating disease severity, as seen in the significant improvement of proptosis and soft tissue inflammation. Furthermore, previous study found that RTX associated with the consumption and resistance of mature B lymphocytes, contributing to control inflammation [25].

Besides CAS, reduction in TRAbs level was observed at 6-month and 12-month observation period after RTX use. However, it was reported that serum TRAbs levels were not changed significantly, and was slightly negatively associated with time during 75-week follow-up [14]. Vannucchi et al. found that serum TRAbs have not reduced obviously in TAO cases before 30 weeks since treatment [17]. Based on the previous limited TAO cases treated by RTX, the pooled changes in current results may because TRAb were associated significantly with TAO clinical activity [26]. During the different disease phase, participants with severe TAO have more serum TRAb levels than those with mild-moderate TAO [27]. Although it was possible to include the involvement of B cells, TRAb and cytokines, the detailed mechanism of decreased TRAb levels was still unknown [28]. In addition, the fluctuation of TSH concentration also existed in short- and long-term observation period.

There was a weakly reduced trend in IL-6, which were around the normal range. However, it was different from the prior studies [29]. IL-6 secreted by diverse cells like T and B lymphocytes, monocytes, and fibroblasts could participate in stimulating T cell, triggering immunoglobulin secretion, also have impacts in fibroblasts and macrophages in TAO [30, 31]. It was likely that blocking IL-6 might be a promising therapy in TAO. But the inapparent results in current meta-analysis could be owing to the small number of participants. Thus large-scale researches were needed in order to make reliable conclusions.

There were several limitations of our review that should be interpreted. First, we have only limited sample size to hardly explore the potential impact of other risk factors such as age, gender, smoking status, dosage of RTX. Second, the orbital variation after RTX treatment could be susceptible to the previous medication of intravenous or oral corticosteroid. Third, subgroup analysis was on the basis of aggregate data, which could mask diversity within individual level and interaction between factors.

In spite of these, the strengths of this review were that we had a systematic search according to prespecified strategies, and tried to find additional studies. We cross-checked methodological decisions and the influence of each study through sensitivity analyses, and showed the robust results, which were similar to those previous trials, making the results more faithworthy in practice in many countries.

## Conclusions

In short, our analysis of existing empirical evidences suggested that the preliminary effects of RTX treatment on TAO might be promising. We initiated the systematic review of RTX therapy of patients with TAO, which tried to fill in knowledge gaps. However, more large-sample and high-quality studies targeting RTX use during this disease and long-term surveillance of prognosis are urgently warranted.

### Additional files

Additional file 1: Data collection forms. (DOC 70 kb)

Additional file 2: Table S1. Quality evaluation of included studies using GRADE criteria. (DOC 70 kb)

Additional file 3: Table S2. Quality evaluation of included studies using NOS. (DOC 38 kb)

Additional file 4: Figure S1. Judgements about each risk of bias item presented as percentages across all included studies. (TIFF 26 kb)

Additional file 5: Figure S2. Judgements about each risk of bias item for each included study. (TIFF 24 kb)

Additional file 6: Table S3. Mean changes for subgroup during RTX for the treatment of TAO. (DOC 46 kb)

## Appendix

**Search strategy:**

Pubmed (1950-present)

1. (rituximab OR RTX)
2. (Graves OR thyroid)
3. (ophthalmopathy OR (eye disease))
4. 1 AND 2 AND 3
5. “rituximab”[Mesh]
6. “Graves ophthalmopathy”[Mesh]
7. 5 AND 6
8. 4 OR 7

Embase(1980-present)

1. ‘rituximab’:ab,ti
2. ‘RTX’:ab,ti
3. 1 OR 2
4. ‘Graves’:ab,ti
5. ‘thyroid’:ab,ti
6. 4 OR 5
7. ‘ophthalmopathy’:ab,ti
8. ‘eye disease’:ab,ti
9. 7 OR 8
10. 3 AND 6 AND 9

Scoups

1. TITLE-ABS-KEY (“rituximab”)
2. TITLE-ABS-KEY (“RTX”)
3. 1 OR 2
4. TITLE-ABS-KEY (“Graves”)
5. TITLE-ABS-KEY (“thyroid”)
6. 4 OR 5
7. TITLE-ABS-KEY (“ophthalmopathy”)
8. TITLE-ABS-KEY (“eye disease”)
9. 7 OR 8
10. 3 AND 6 AND 9

## Abbreviations

CAS: Clinical activity score
CI: Confidence interval
GD: Graves’ disease
GRADE: Grading of recommendations assessment, development and evaluation
IL-6: Interleukin-6
NOS: Newcastle-Ottawa scale
PRISMA: Preferred reporting items for systematic reviews and meta-analyses
RTX: Rituximab
SMD: Standard mean difference
TAO: Thyroid-associated ophthalmopathy
TRAb: Thyrotropin receptor antibody
TSH: Thyroid stimulating hormone
