# Supplementary material for: Does rituximab improve clinical outcomes of patients with thyroid-associated ophthalmopathy? A systematic review and meta-analysis
Source: BMC Ophthalmol. 2018 Feb 17;18:46. doi: 10.1186/s12886-018-0679-4 (PMC5816536; doi:10.1186/s12886-018-0679-4)
Supplement: Supplementary file 1 — Data collection forms. (DOC 70 kb) [file 12886_2018_679_MOESM1_ESM.doc]

**Additional file 1**

**Data collection forms**

**1) The primary outcome**

| ID | Initial CAS | | | Post-RTX 1-month CAS | | |
| --- | --- | --- | --- | --- | --- | --- |
|  | Mean0 | SD0 | n0 | Mean1 | SD1 | n1 |
| 1 |  |  |  |  |  |  |
| 2 |  |  |  |  |  |  |

| ID | Initial CAS | | | Post-RTX 3-month CAS | | |
| --- | --- | --- | --- | --- | --- | --- |
|  | Mean0 | SD0 | n0 | Mean1 | SD1 | n1 |
| 1 |  |  |  |  |  |  |
| 2 |  |  |  |  |  |  |

| ID | Initial CAS | | | Post-RTX 6-month CAS | | |
| --- | --- | --- | --- | --- | --- | --- |
|  | Mean0 | SD0 | n0 | Mean1 | SD1 | n1 |
| 1 |  |  |  |  |  |  |
| 2 |  |  |  |  |  |  |

| ID | Initial CAS | | | Post-RTX 12-month CAS | | |
| --- | --- | --- | --- | --- | --- | --- |
|  | Mean0 | SD0 | n0 | Mean1 | SD1 | n1 |
| 1 |  |  |  |  |  |  |
| 2 |  |  |  |  |  |  |

**2) The secondary outcomes**

| ID | Initial proptosis | | | At least 1-month proptosis | | |
| --- | --- | --- | --- | --- | --- | --- |
|  | Mean0 | SD0 | n0 | Mean1 | SD1 | n1 |
| 1 |  |  |  |  |  |  |
| 2 |  |  |  |  |  |  |

| ID | Initial TRAbs | | | At least 6-month TRAbs | | |
| --- | --- | --- | --- | --- | --- | --- |
|  | Mean0 | SD0 | n0 | Mean1 | SD1 | n1 |
| 1 |  |  |  |  |  |  |
| 2 |  |  |  |  |  |  |

| ID | Initial TRAbs | | | At least 12-month TRAbs | | |
| --- | --- | --- | --- | --- | --- | --- |
|  | Mean0 | SD0 | n0 | Mean1 | SD1 | n1 |
| 1 |  |  |  |  |  |  |
| 2 |  |  |  |  |  |  |

| ID | Initial TSH | | | At least 3-month TSH | | |
| --- | --- | --- | --- | --- | --- | --- |
|  | Mean0 | SD0 | n0 | Mean1 | SD1 | n1 |
| 1 |  |  |  |  |  |  |
| 2 |  |  |  |  |  |  |

| ID | Initial TSH | | | At least 12-month TSH | | |
| --- | --- | --- | --- | --- | --- | --- |
|  | Mean0 | SD0 | n0 | Mean1 | SD1 | n1 |
| 1 |  |  |  |  |  |  |
| 2 |  |  |  |  |  |  |

| ID | Initial IL-6 | | | At least 6-month IL-6 | | |
| --- | --- | --- | --- | --- | --- | --- |
|  | Mean0 | SD0 | n0 | Mean1 | SD1 | n1 |
| 1 |  |  |  |  |  |  |
| 2 |  |  |  |  |  |  |
