# Supplementary material for: Does rituximab improve clinical outcomes of patients with thyroid-associated ophthalmopathy? A systematic review and meta-analysis
Source: BMC Ophthalmol. 2018 Feb 17;18:46. doi: 10.1186/s12886-018-0679-4 (PMC5816536; doi:10.1186/s12886-018-0679-4)
Supplement: Supplementary file 2 — Quality evaluation of included studies using GRADE criteria. (DOC 70 kb) [file 12886_2018_679_MOESM2_ESM.doc]

Additional file 2: Table S1 Quality evaluation of included studies using GRADE criteria*

| Comparator  (before RTX) | Intervention  (after RTX) | No of Participants (studies) | Type of study | Follow up (months) | SMD (95%CI) with initial values | Quality of the evidence (GRADE) |
| --- | --- | --- | --- | --- | --- | --- |
| initial CAS | post-RTX 1-month CAS | 60 (4 studies) | Cohort study | 12 to 60 | **1.88 to 14.2** | ⊕⊝⊕⊕ Moderate  ●High inconsistency |
| initial CAS | post-RTX 3-month CAS | 52 (3 studies) | Cohort study | 12 to 18 | **1.33 to 4.63** | ⊕⊝⊕⊕ Moderate  ●High inconsistency |
| initial CAS | post-RTX 3-month CAS | 30 (1 study) | Randomized controlled trial | 19 | **5.44 (3.84 to 7.03)** | ⊕⊕⊕⊝ Moderate  ●Publication bias |
| initial CAS | post-RTX 6-month CAS | 72 (4 studies) | Cohort study | 1 to 16 | **1.33 to 6** | ⊕⊝⊕⊕ Moderate  ●High inconsistency |
| initial CAS | post-RTX 6-month CAS | 206 (3 studies) | Randomized controlled trial | 10 to 19 | **0.79 to 5.2** | ⊕⊕⊕⊕High |
| initial CAS | post-RTX 12-month CAS | 52 (3 studies) | Cohort study | 12 to 60 | **1.9 to 5.85** | ⊕⊝⊕⊕ Moderate  ●High inconsistency |
| initial CAS | post-RTX 12-month CAS | 26 (1 study) | Randomized controlled trial | 13 | **2.08 (1.11 to 3.05)** | ⊕⊕⊕⊝ Moderate  ●Publication bias |
| initial proptosis | at least 1-month proptosis | 40 (3 studies) | Cohort study | 1 to 18 | **0.11 to 2.72** | ⊕⊝⊕⊝ Low  ●High inconsistency, publication bias |
| initial proptosis | at least 1-month proptosis | 176 (2 studies) | Randomized controlled trial | 10 to 13 | -0.14 to 1.31 | ⊕⊝⊕⊝ Low  ●High inconsistency, publication bias |
| initial TRAbs | post-RTX 6-month TRAbs | 38 (2 studies) | Cohort study | 1 to 12 | **0.97 to 1.32** | ⊕⊕⊝⊕ Moderate  ●Imprecision |
| initial TRAbs | post-RTX 6-month TRAbs | 56 (2 studies) | Randomized controlled trial | 13 to 19 | **0.3 to 0.93** | ⊕⊕⊕⊝ Moderate  ●Publication bias |
| initial TRAbs | post-RTX 12-month TRAbs | 10 (1 study) | Cohort study | 60 | **2.21 (0.56 to 3.85)** | ⊝⊕⊕⊝ Low  ●Risk of bias, publication bias |
| initial TRAbs | post-RTX 12-month TRAbs | 30 (1 study) | Randomized controlled trial | 19 | **1.36 (0.56 to 2.16)** | ⊕⊕⊕⊝ Moderate  ●Publication bias |
| initial TSH | post-RTX 3-month TSH | 24 (1 study) | Cohort study | 12 | **1.12 (0.26 to 1.99)** | ⊝⊕⊕⊝ Low  ●Risk of bias, publication bias |
| initial TSH | post-RTX 3-month TSH | 30 (1 study) | Randomized controlled trial | 19 | **0.39 (0.33 to 1.11)** | ⊕⊕⊕⊝ Moderate  ●Publication bias |
| initial TSH | post-RTX 12-month TSH | 34 (1 study) | Cohort study | 12 to 60 | -0.87 to 0.38 | ⊝⊕⊕⊝ Low  ●Risk of bias, publication bias |
| initial TSH | post-RTX 12-month TSH | 30 (1 study) | Randomized controlled trial | 19 | **1.36 (0.56 to 1.26)** | ⊕⊕⊕⊝ Moderate  ●Publication bias |
| initial IL-6 | post-RTX 6-month IL-6 | 20 (1 study) | Cohort study | 12 | **1.52 (0.51 to 2.52)** | ⊕⊝⊕⊝ ●High inconsistency, publication bias |
| initial IL-6 | post-RTX 6-month IL-6 | 150 (1 study) | Randomized controlled trial | 10 | **11.45 (10.11 to 12.79)** | ⊕⊕⊕⊝ Moderate  ●Publication bias |
|  |  |  |  |  |  |  |

RTX=rituximab;

SMD = standardized mean difference;

CAS=clinical activity score;

TRAb=thyrotropin receptor antibody;

TSH=thyroid stimulating hormone;

IL-6=interleukin-6;

● =Reason for quality of the evidence

GRADE: Grading of Recommendations Assessment, Development and Evaluation

*The results included in the Table S1 were based on the assumption of a consistent mean differences. The implications of this effect for populations were considered at different baseline risks. Based on the assumed differences, corresponding differences after RTX treatment were estimated using the meta-analytic standard mean difference.
