# Supplementary material for: Does rituximab improve clinical outcomes of patients with thyroid-associated ophthalmopathy? A systematic review and meta-analysis
Source: BMC Ophthalmol. 2018 Feb 17;18:46. doi: 10.1186/s12886-018-0679-4 (PMC5816536; doi:10.1186/s12886-018-0679-4)
Supplement: Supplementary file 3 — Quality evaluation of included studies using NOS. (DOC 38 kb) [file 12886_2018_679_MOESM3_ESM.doc]

Additional file 3: Table S2 Quality evaluation of included studies using NOS.

| Study | Selection |  |  |  | Comparability | Exposure |  |  |  |
| --- | --- | --- | --- | --- | --- | --- | --- | --- | --- |
|  | Adequate definition of cases | Representativeness of cases | Selection of controls | Deﬁnition of controls | Comparability of cases and controls on the basis of the design or analysis | Ascertainment of exposure | Same method of ascertainment for cases and controls | Nonresponse rate | Total scores |
| Salvi M et al. 2007 | ★ | ★ | ★ | ★ | ★ | 0 | ★ | ★ | 7 |
| Vannucchi G et al. 2010 | ★ | 0 | ★ | ★ | ★ | 0 | ★ | ★ | 6 |
| Silkiss RZ et al. 2010 | ★ | ★ | ★ | ★ | ★ | 0 | ★ | ★ | 7 |
| Khanna D et al. 2010 | ★ | ★ | ★ | ★ | ★ | 0 | ★ | ★ | 7 |
| Mitchell AL et al. 2013 | ★ | ★ | ★ | ★ | ★ | 0 | ★ | 0 | 6 |
| Savino G et al. 2013 | ★ | ★ | ★ | ★ | ★ | 0 | ★ | ★ | 7 |
| Erdei A et al. 2014 | ★ | ★ | ★ | ★ | ★ | 0 | ★ | ★ | 7 |
| McCoy AN et al. 2014 | ★ | ★ | ★ | ★ | ★ | 0 | ★ | ★ | 7 |

NOS= Newcastle-Ottawa quality assessment score

★The star indicates a score of 1.
