# Supplementary figures and images for: Does rituximab improve clinical outcomes of patients with thyroid-associated ophthalmopathy? A systematic review and meta-analysis
Source: BMC Ophthalmol. 2018 Feb 17;18:46. doi: 10.1186/s12886-018-0679-4 (PMC5816536; doi:10.1186/s12886-018-0679-4)

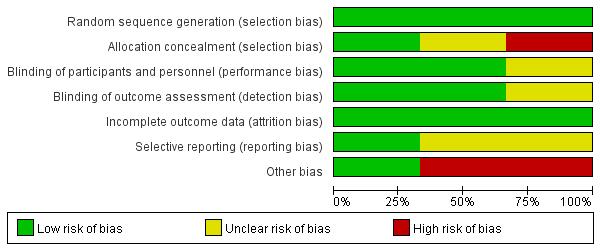

Supplement: Supplementary file 4 — Judgements about each risk of bias item presented as percentages across all included studies. (TIFF 26 kb) [file 12886_2018_679_MOESM4_ESM.tif]

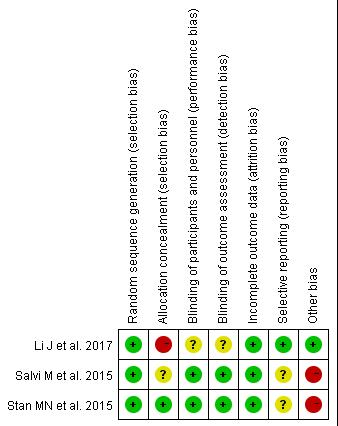

Supplement: Supplementary file 5 — Judgements about each risk of bias item for each included study. (TIFF 24 kb) [file 12886_2018_679_MOESM5_ESM.tif]
