# Supplementary material for: Does rituximab improve clinical outcomes of patients with thyroid-associated ophthalmopathy? A systematic review and meta-analysis
Source: BMC Ophthalmol. 2018 Feb 17;18:46. doi: 10.1186/s12886-018-0679-4 (PMC5816536; doi:10.1186/s12886-018-0679-4)
Supplement: Supplementary file 6 — Mean changes for subgroup during RTX for the treatment of TAO. (DOC 46 kb) [file 12886_2018_679_MOESM6_ESM.doc]

Additional file 6: Table S3 Mean changes for subgroup during RTX for the treatment of TAO

| Subgroup | Number of studies | SMD (95%CI) | Heterogeneity test | |  |  | Publication bias | |
| --- | --- | --- | --- | --- | --- | --- | --- | --- |
|  |  |  | *Q* | *τ^2^* | *I^2^(%)* | *p* | *t* | *p* |
| CAS |  |  |  |  |  |  |  |  |
| 1 month | 4 | 4.41(1.87-6.95) | 19.17 | 5.1033 | 84.4 | <0.001 | 2.26 | 0.152 |
| 3 month | 4 | 3.89(1.67-6.11) | 23.9 | 4.3534 | 87.4 | <0.001 | 1.68 | 0.236 |
| 6 month | 7 | 3.59(1.83-5.35) | 106.16 | 5.0124 | 94.3 | <0.001 | 0.51 | 0.633 |
| 12 month | 4 | 3.04(1.58-4.50) | 13.17 | 1.6858 | 77.2 | 0.004 | 1.5 | 0.273 |
| Proptosis |  |  |  |  |  |  |  |  |
| at least 1 month | 5 | 0.97(0.10-1.84) | 19.97 | 0.7287 | 80 | <0.001 | -0.33 | 0.763 |
| TRAbs |  |  |  |  |  |  |  |  |
| 6 month | 4 | 0.82(0.40-1.25) | 2.88 | NA | 0 | 0.41 | 1.15 | 0.37 |
| 12 month | 2 | 1.52(0.80-2.24) | 0.83 | NA | 0 | 0.363 | NA | NA |
| TSH |  |  |  |  |  |  |  |  |
| 3 month | 2 | 0.69(0.14-1.25) | 1.63 | NA | 38.5 | 0.202 | NA | NA |
| 12 month | 3 | 0.39(-0.74-1.52) | 8.59 | 0.7518 | 76.7 | 0.014 | -1.49 | 0.376 |
| IL-6 |  |  |  |  |  |  |  |  |
| 6 month | 2 | 6.47(-3.26-16.21) | 134.47 | 48.9543 | 99.3 | <0.001 | NA | NA |

SMD = standardized mean difference;

CAS=clinical activity score;

TRAb=thyrotropin receptor antibody;

TSH=thyroid stimulating hormone;

IL-6=interleukin-6;

NA=not available

In this study, heterogeneity was appraised through *Q*, *t^2^*, and I2 statistics. *Q* value means random error; *t^2^* value means the variation between studies; *I^2^* value means the ratio of inter-study variation within the total heterogeneity.
